# Clinical correlates of anti-SARS-CoV-2 antibody profiles in Spanish COVID-19 patients from a high incidence region

**DOI:** 10.1038/s41598-021-83969-5

**Published:** 2021-02-23

**Authors:** Robert Markewitz, Antje Torge, Klaus-Peter Wandinger, Daniela Pauli, Andre Franke, Luis Bujanda, José Maria Marimón, Jesus M. Banales, María A. Gutierrez-Stampa, Beatriz Nafría, Ralf Junker

**Affiliations:** 1 Institute of Clinical Chemistry, University Hospital Schleswig-Holstein, Kiel/Lübeck, Germany; 2 Institute of Clinical Molecular Biology, Christian-Albrechts-University of Kiel & University Hospital Schleswig-Holstein, Kiel, Germany; 3 Department of Liver and Gastrointestinal Diseases, Biodonostia Health Research Institute - Donostia University Hospital, San Sebastián, Spain; 4 National Institute for the Study of Liver and Gastrointestinal Diseases (CIBERehd, “Instituto de Salud Carlos III”), University of the Basque Country (UPV/EHU), San Sebastián, Spain; 5 Infectious Diseases Area, Respiratory Infection and Antimicrobial Resistance Group, Microbiology Department, Biodonostia Health Research Institute, Osakidetza Basque Health Service, Donostialdea Integrated Health Organisation, San Sebastián, Spain; 6 Ikerbasque, Basque Foundation for Science, Bilbao, Spain; 7 Osakidetza, OSI Donostialdea, Altza Primary Care, Biodonostia Health Research Institute, San Sebastián, Spain; 8 Clinical Biochemistry Department, Biodonostia Health Research Institute Osakidetza Basque Health Service, Donostialdea Integrated Health Organisation, San Sebastián, Spain

**Keywords:** Viral infection, SARS-CoV-2, Diagnostic markers

## Abstract

Laboratory testing for the severe acute respiratory syndrome coronavirus 2 (SARS-CoV-2) consists of two pillars: the detection of viral RNA via rt-PCR as the diagnostic gold standard in acute cases, and the detection of antibodies against SARS-CoV-2. However, concerning the latter, questions remain about their diagnostic and prognostic value and it is not clear whether all patients develop detectable antibodies. We examined sera from 347 Spanish COVID-19 patients, collected during the peak of the epidemic outbreak in Spain, for the presence of IgA and IgG antibodies against SARS-CoV-2 and evaluated possible associations with age, sex and disease severity (as measured by duration of hospitalization, kind of respiratory support, treatment in ICU and death). The presence and to some degree the levels of anti-SARS-CoV-2 antibodies depended mainly on the amount of time between onset of symptoms and the collection of serum. A subgroup of patients did not develop antibodies at the time of sample collection. Compared to the patients that did, no differences were found. The presence and level of antibodies was not associated with age, sex, duration of hospitalization, treatment in the ICU or death. The case-fatality rate increased exponentially with older age. Neither the presence, nor the levels of anti-SARS-CoV-2 antibodies served as prognostic markers in our cohort. This is discussed as a possible consequence of the timing of the sample collection. Age is the most important risk factor for an adverse outcome in our cohort. Some patients appear not to develop antibodies within a reasonable time frame. It is unclear, however, why that is, as these patients differ in no respect examined by us from those who developed antibodies.

## Introduction

Currently the world is facing a pandemic of the coronavirus disease 2019 (COVID-19), caused by the severe acute respiratory syndrome coronavirus 2 (SARS-CoV-2), first detected in Wuhan, China in December 2019^1^. While some infections are associated with an asymptomatic clinical course, the spectrum of illness severity among symptomatic infections is wide, ranging from mild cases to critical ones with respiratory failure, or dysfunction of multiple other organ systems^2–6^. The proportion of severe cases as well as the case-fatality rate (CFR) vary widely between different geographic locations, possibly due to different demographics and approaches to testing for SARS-CoV-2^7^.

The hallmark of laboratory testing for SARS-CoV-2 in suspected acute cases of COVID-19 is the detection of viral RNA via real-time polymerase chain reaction (rt-PCR)^8^. Over time, different assays for the detection of antibodies against different target antigens of SARS-CoV-2 have also become available. While it is widely assumed that antibodies against SARS-CoV-2 can be used as a marker for a past or present infection with SARS-CoV-2 and that they are a marker of immunity against it, many questions surrounding them have not been answered definitively:

- Do all patients with PCR-confirmed infections with SARS-CoV-2 develop antibodies, and if not, what does that mean for the patients who do or do not develop antibodies? One recent study suggested that up to 30% of patients do not develop antibodies after a PCR-confirmed infection with SARS-CoV-2^9^.
- Is the development of antibodies helpful to eradicate the virus or do they even cause harm via immunopathologic mechanisms? There have been recent considerations concerning the possibility of antibody-dependent enhancement of SARS-CoV-2^10^
- Can antibodies against SARS-CoV-2 be used as a prognostic marker? And if yes, do they improve or worsen a patient’s prognosis?

In order to answer some of these questions, we examined sera from a cohort of Spanish COVID-19 patients from a high incidence region, all of whom had PCR-confirmed infections with SARS-CoV-2, for the presence of antibodies against SARS-CoV-2. Specifically, we wanted to investigate whether the presence of anti-SARS-CoV-2 antibodies can be used as a marker of disease severity and what factors correlate with the presence of antibodies against SARS-CoV-2.

## Methods

Serum samples from 347 Spanish patients from a high-incidence region were collected at one point in time (ranging from 0 to 33 days since onset of symptoms) during their stay at the Hospital Universitario Donostia (Donostia/San Sebastian, Spain) after they were confirmed to be SARS-CoV-2 positive via rt-PCR, which was performed at the Microbiology Service of the Hospital Universitario Donostia. For the rt-PCR, assays from three different manufacturers were used: Allplex 2019-nCoV Assay (Seegene Inc, Seoul, South Korea), LightMix Modular SARS-CoV-2 (E- gene and RdRP gene) Assay (TibMolbiol, Berlin, Germany) and Viasure SARS-CoV-2 (ORF1ab and N genes) assay (Certest Biotec, Zaragoza, Spain). Antibody testing was performed at the Institute of Clinical Chemistry, at the University Hospital Schleswig–Holstein (Kiel/Lübeck, Germany). The sera were examined for the presence of IgA and IgG antibodies against the Spike-protein (S1-subunit, including the receptor binding domain (RBD)) of SARS-CoV-2 using the Anti-SARS-CoV-2-ELISA kits from EUROIMMUN (Lübeck, Germany) according to the manufacturers’ instructions. This test yields a (dimensionless) ratio of the optical density (OD) of the sample at 450 nm compared to the OD of a calibrator. Ratios of ≥ 0.8 to < 1.1 are considered borderline, ratios of ≥ 1.1 are considered positive. According to the manufacturers’ specifications, testing for anti-SARS-CoV-2 IgA after more than ten days since onset of symptoms has a sensitivity of 98.6% and a specificity of 92.0%, testing for IgG a sensitivity of 94.4% and a specificity of 99.6%. The S1-subunit of the Spike-protein was chosen as target antigen because of evidence that antibodies against the Spike-protein, especially the RBD, are neutralizing and therefore potentially confer immunity^11–13^.

Further, the patients’ age and sex were recorded, as was the amount of days that elapsed between the onset of symptoms and the collection of the serum sample. Also, as markers of disease severity and outcome, the duration of the hospital stay, treatment in the ICU, the kind of respiratory support the patient received and death were evaluated.

The study was approved of by the Ethics Committee for Clinical Research of Euskadi (CEIC-E) (PI2020064). All participants gave written informed consent to all procedure they underwent. For minors under the age of 18 years, this consent was obtained from the patients’ legally authorized representatives. For patients who died in the course of the disease or who were too ill to consent, this consent was also obtained from legally authorized persons as mandated by the local ethics committee. The study and all of its’ procedures were conducted in concordance with the declaration of Helsinki.

### Statistics

Associations between continuous variables were tested for via calculation of the correlation coefficients according to Pearson and Spearman. These results were visualized through a scatterplot for which linear regressions as well as their slopes and intercepts were calculated. Differences between groups were tested for statistical significance via ANOVA and visualized as combined box and dot plots. Differences were treated as statistically significant for p-values ≤ 0.05. Levels of significance were indicated in all relevant figures as follows: ns: p > 0.05; *: p ≤ 0.05; **: p ≤ 0.01; ***: p ≤ 0.001; ****: p ≤ 0.0001. All data files were processed in the free software for statistical computing and graphics R (version 4.0.3) with the integrated development environment RStudio (version 1.3.1093)^14^.

### Ethics approval

The study was approved of by the Ethics Committee for Clinical Research of Euskadi (CEIC-E) (PI2020064).

### Consent to participate

All participants gave written informed consent to all procedure they underwent. The study was conducted in concordance with the declaration of Helsinki.

## Results

### Sample characteristics

We examined serum samples from 347 patients who were treated at the Hospital Universitario Donostia in Donostia/San Sebastian, Spain between March 26th and April 11th and who were infected with SARS-CoV-2 as confirmed via PCR. Their mean age was 64.3 years (± 14.1; range: 10–95 years). Of the 347 patients, 144 (41.5%) were female and 203 (58.5%) were male. The mean duration of their hospital stay was 14.3 days (± 14.4; range: 1–79 days). During their stay at the hospital, 54 patients (15.6%) were treated in the ICU and 38 (11%) died. Moreover, 15 patients (4.3%) received no respiratory support, 281 (81%) received O_2_ non-invasively and 51 (14.7%) received invasive ventilation. Of the patients who needed treatment in the ICU, 13 (24.1%) died. Similarly, of those who needed invasive ventilation the same 13 (25.5%) died. For an overview of the patient and sample characteristics, see Table 1.

**Table 1:** Overview of the patient characteristics; each absolute number is also represented as percentage of group defined by the column.

	All patients	Male	female	ICU	Deceased
Male	203 (58.8%)	–	–	39 (72.2%)	21 (55.3%)
Female	144 (41.5%)	–	–	15 (27.8%)	17 (44.7%)
ICU	54 (15.6%)	39 (19.2%)	15 (10.4%)	–	13 (34.2%)
Decease	38 (11%)	21 (10.3%)	17 (11.8%)	13 (24.1%)	–

**Figure 1:**
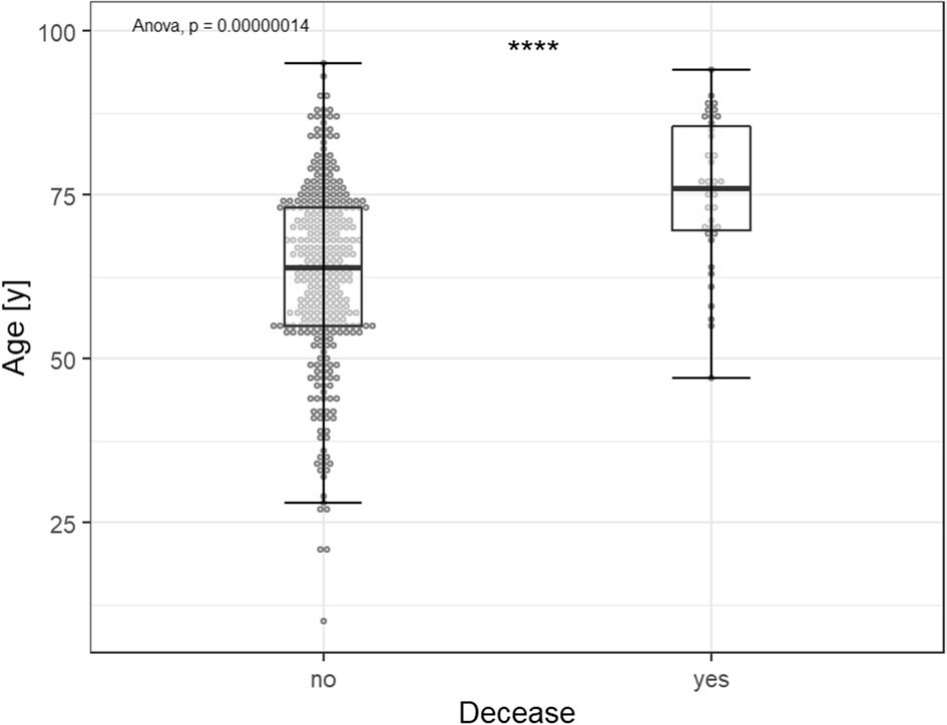
Comparison of the mean age of deceased and surviving patients.

**Figure 2:**
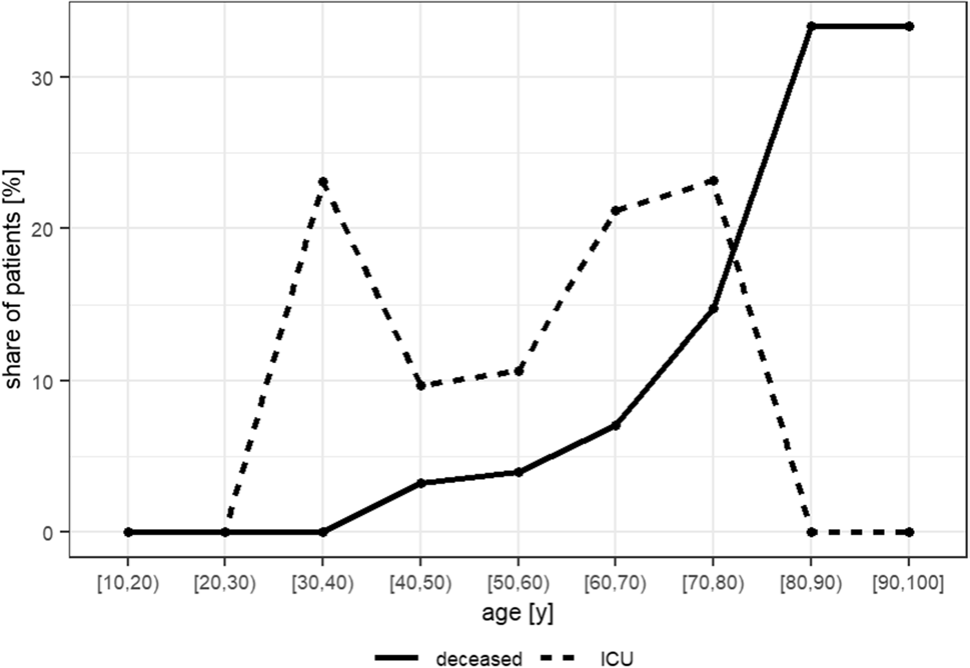
Case fatality rate (line “deceased”) and share of patients treated in the ICU (line “ICU”) visualized for each different life-decade seperately.

There was a statistically significant association between age and survival, the mean age of deceased patients (75.2 years; ± 10.9) being significantly higher than the mean age of surviving patients (63 years; ± 13.8; p < 0.001; see Fig. 1). Furthermore, the CFR rises near-exponentially for each life-decade from 3.2% for 40–49 year old patients to 32.6% for > 80 year old patients (see Fig. 2). Conversely, 89.5% of those who died were 60 years of age or older. No such relationship was observed for either age or treatment in the ICU or age and duration of hospitalization.

### Serologic results

**Figure 3:**
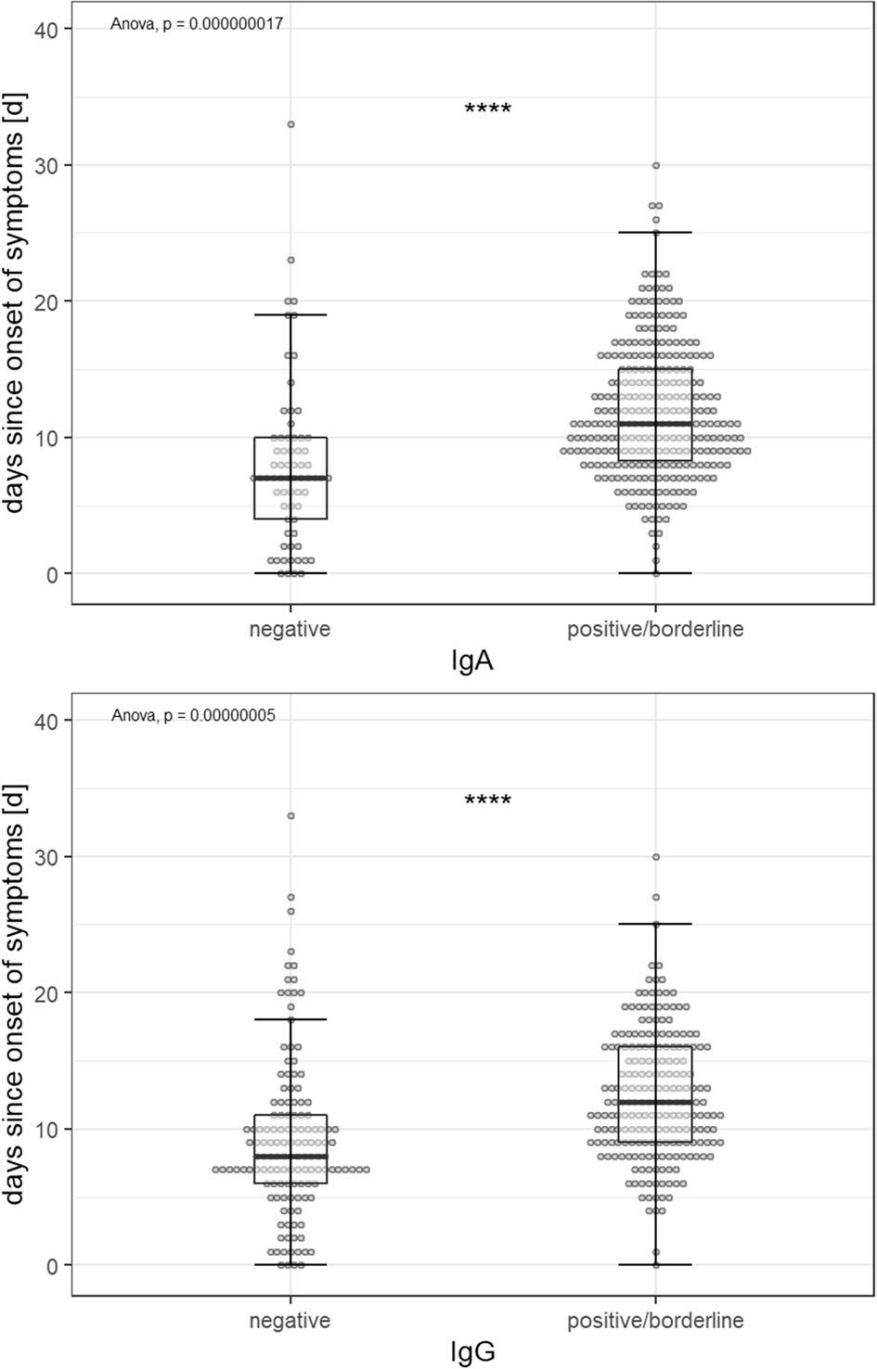
Comparison of the mean amount of days from symptom onset until sample collection between negative and borderline/positive samples for IgA (top) and IgG (bottom).

**Figure 4:**
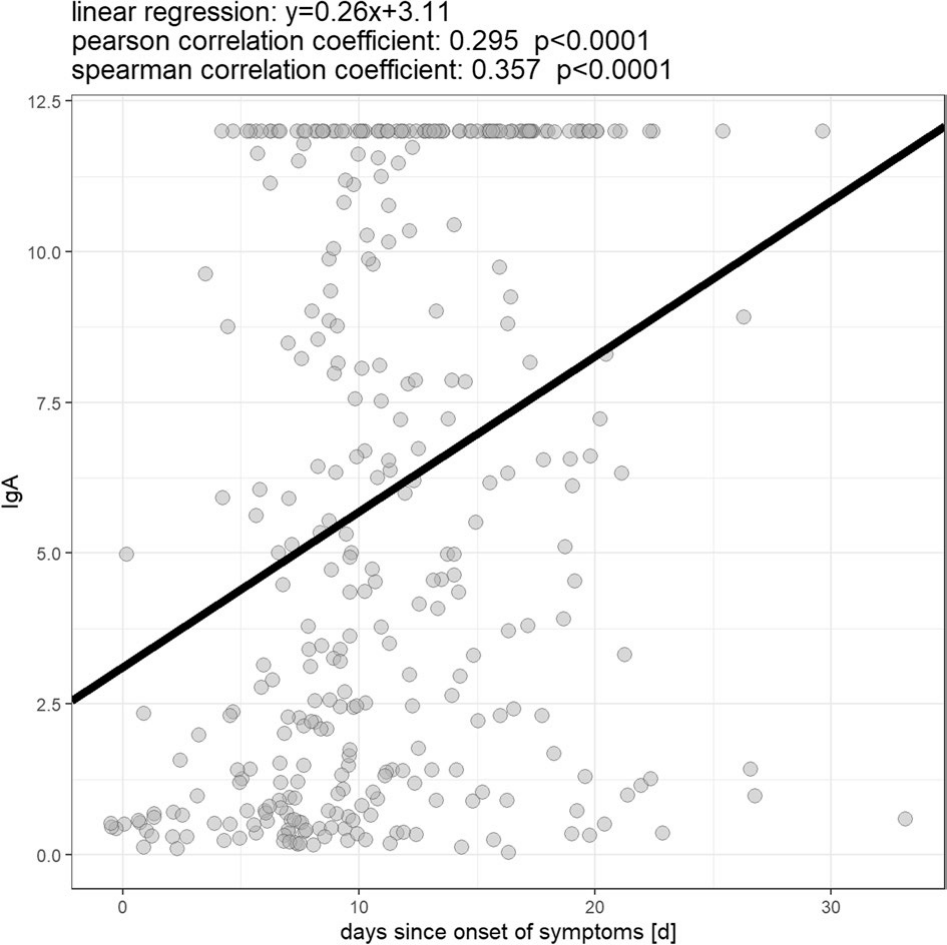
Correlation between the amount of days since onset of symptoms and IgA antibody level.

**Figure 5:**
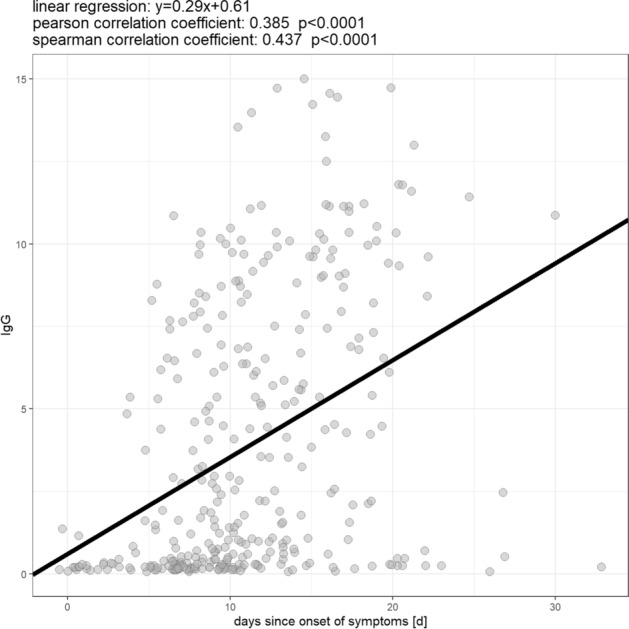
Correlation between the amount of days since onset of symptoms and IgG antibody level.

**Figure 6:**
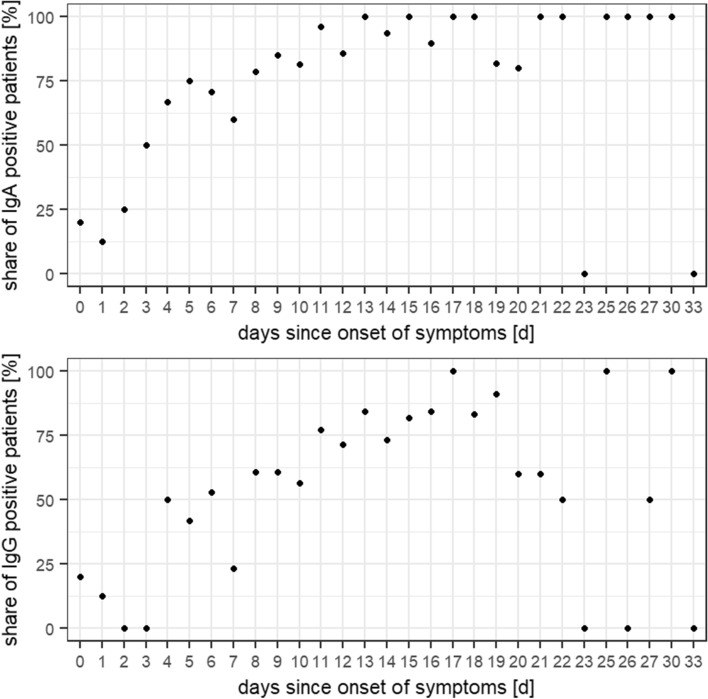
Share of samples collected on different days since onset of symptoms that were positive for IgA (top), IgG (middle) or either of those (bottom).

Of the 347 samples, 282 (81.3%) were positive or borderline for IgA and/or IgG antibodies against the Spike-protein of SARS-CoV-2 and 65 (18.7%) were overall negative. For a complete overview of the numbers and shares of positive samples see Table 2. Sera in which antibodies against SARS-CoV-2 could be detected differed from negative sera mainly in one respect: positive samples were collected after a significantly longer amount of days since onset of symptoms than negative ones. This is true for both the detection of IgA (mean amount of days between onset of symptoms and sample collection: 11.9 days (IgA positive; ± 4.9 days; range: 0–30 days) vs. 7·9 days (IgA negative; ± 6.1 days; range: 0–33 days; p < 0.001) and IgG (12.4 days (IgG positive; ± 4.7 days; range: 0–30 days) vs. 9.2 days (IgG negative; ± 5.8 days; range: 0–33 days); p < 0.001; see Fig. 3). For both IgA and IgG positive samples, there is also a statistically significant correlation of medium effect size between the amount of days since onset of symptoms and the level of the measured OD ratio (IgA: Spearman correlation coefficient: 0.357; p < 0.001; IgG: Spearman correlation coefficient: 0.437; p < 0.001; see Figs. 4 and 5). If samples collected on the same day since onset of symptoms are considered collectively, the percentage of IgA and IgG positive samples from each day after onset of symptoms describes a steady upward curve until it reaches a first maximum of 100% positive samples after 13 days for IgA and 17 days for IgG (see Fig. 6). It is notable, however, that even after this point there is a small but persistent amount of samples that remains seronegative (IgG: 14 negative samples (4.0%) after day 17 since onset of symptoms; IgA: 9 negative samples (2.6%) after day 13 since onset of symptoms) after day 13 since onset of symptoms), most prominently one sample collected 33 days since onset of symptoms, which is IgA and IgG negative.

**Table 2:** Overview of numbers and shares of patients who were tested borderline (bdl.) or positive (pos.) for one or both anti-SARS-CoV-2 antibody (ab) classes within the whole cohort and in distinct subpopulations.

	IgA bdl	IgA pos	IgA bdl./pos	IgG bdl	IgG pos	IgG bdl./pos	Any ab bdl./pos
All patients	16 (4.6%)	263 (75.8%)	279 (80.4%)	17 (4.9%)	194 (55.9%)	211 (60.8%)	282 (81.3%)
Male	8 (3.9%)	159 (78.3%)	167 (82.3%)	14 (6.9%)	116 (57.1%)	130 (64%)	168 (82.8%)
Female	8 (5.6%)	104 (72.2%)	112 (77.8%)	3 (2.1%)	78 (54.2%)	81 (56.3%)	114 (79.2%)
ICU	0	51 (94.4%)	51 (94.4%)	2 (3.7%)	43 (79.6%)	45 (83.3%)	51 (94.4%)
Deceased	0	25 (65.8%)	25 (65.8 5)	2 (5.3%)	18 (47.4%)	20 (52.6%)	26 (68.4%)

Furthermore, there was an association of medium effect size between the detection of IgA and respiratory support with non-invasive O_2_ (Cramer’s V: 0.31) and the detection of any antibody (IgA and/or IgG) and respiratory support with non-invasive O_2_ (Cramer’s V: 0.32).

**Figure 7:**
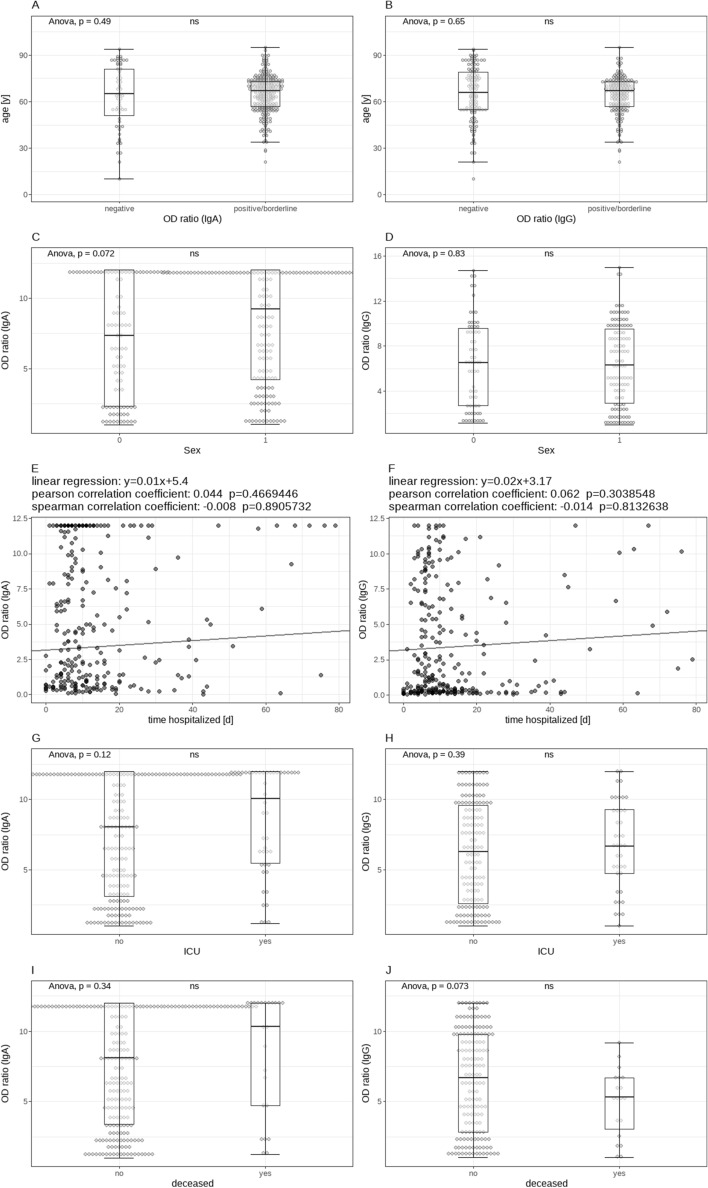
Visualization of the relationship between the detection of anti-SARS-CoV-2 antibodies and age (**A**,**B**), sex (**C**,**D**), duration of hospitalization (**E**,**F**), treatment in the ICU (**G**,**H**) and death (**I**,**J**) for all samples of our cohort. No statistically significant associations could be found for any of these variables.

Anti-SARS-CoV-2 positive and negative patients did not differ significantly in age, gender or the duration of their hospitalization. There was also no significant association between the serologic status and treatment in an ICU or death. For a visualization of these relationships, along with their levels of significance, see Fig. 7. Furthermore, only taking into account positive samples, there was no statistically significant difference in the measured OD ratio levels between patients who were or were not treated in the ICU, between the different types of respiratory support or between patients who did or did not die. There was also no correlation between the OD ratio and age.

**Figure 8:**
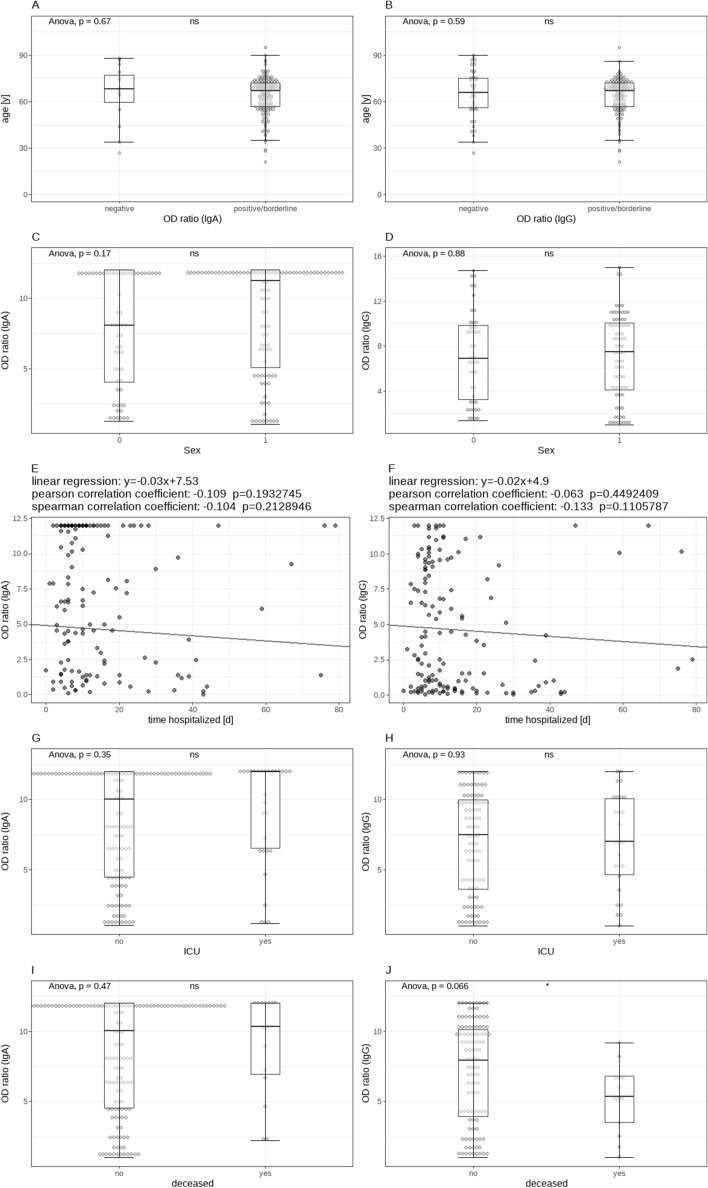
Visualization of the same analyses as in Fig. 7 for all samples collected at least ten days after onset of symptoms. Again, no statistically significant associations could be found.

Since there was a statistically significant impact on the serologic status of the point in time since onset of symptoms at which the serum sample was collected, the same statistical analyses were also run for all samples that were collected at least ten days since onset of symptoms (which is the amount of time for which the test properties are defined by the manufacturer) only. In this subgroup, no associations were found between age, duration of hospitalization, treatment in the ICU or death and the serologic status or the value of measured the OD ratio. For a visualization of these relationships, see Fig. 8. Of note, the association between the detection of antibodies and respiratory support with O_2_ only was not observed for the samples collected at least ten days since onset of symptoms. Of all these samples, 17 (8.5%) remained negative for both IgG and IgA.

## Discussion

Our results demonstrate that the anti-SARS-CoV-2 antibody status strongly depends on the time since symptom onset at which the serologic sample is collected. If serum is collected too early after symptom onset, the sensitivity of the test is considerably decreased. Likewise, at least for IgG, the level of the OD ratio correlates with the amount of time since symptom onset. This finding is not surprising as it corroborates findings of other studies that the sensitivity of tests for antibodies against SARS-CoV-2 depends on the time since onset of symptoms and is especially low in the first week^15,16^. It also underscores the important message that the detection of antibodies is not suitable for the diagnosis of early stages of COVID-19. Serologic examinations should be ordered at the earliest after the amount of time that is recommended in the manufacturers’ instructions, ideally after two to three weeks since symptom onset in order to produce meaningful results.

It is important to note, however, that our results do not indicate that less severe cases of COVID-19 show a lower seroprevalence or lower levels of anti-SARS-CoV-2 antibodies, as has been speculated^15^. At least in our cohort, sensitivity of antibody testing was independent of disease severity.

Our results also show that there are some patients who didn’t develop antibodies even after a sufficient amount of time. However, those patients do not differ in any phenotype that was examined by us from anti-SARS-CoV-2 positive patients. Similar observations have been made before^9^, but only in a cohort with cases of mostly mild to moderate severity. Gudbjartsson et el. also report that even three months after recovery from a PCR-confirmed infection with SARS-CoV-2, 3.9% of the examined patients remained seronegative for antibodies of all classes against both the S- and the N-protein of SARS-CoV-2^17^. This observation could be due to several causes: (1) the PCR that confirmed the infection with SARS-CoV-2 was false-positive, (2) the antibodies developed by the patients were directed against another target antigen, (3) some patients might have developed a mucosal antibody response in the absence of a systemic response or (4) some patients actually don’t develop antibodies against SARS-CoV-2. The first scenario, while theoretically possible, is highly unlikely to account for all seronegative cases, given the high specificity of PCR-testing for SARS-CoV-2. The second scenario reveals testing for only one target antigen as one of the limitations of our study. Of note however, studies examining the antibody responses against different target antigens generally find a good concordance between these responses, making this explanation somewhat less likely^17,18^. Furthermore, the focus on antibodies against the S1-subunit of the spike protein seems prudent to us, as this is also the epitope of neutralizing antibodies (as mentioned above). The third scenario falls within the scope of the possible since it has been shown that mucosal antibody responses against SARS-CoV-2 can occur in the absence of a systemic response (and even in PCR-negative, exposed persons)^19^. It has to be noted, though, that this constellation was mainly observed in asymptomatic cases, whereas our cohort consisted of hospitalized patients, for whom the generally good correlation between the mucosal and the systemic response, that has otherwise been observed^18,19^, seems more likely to be applicable. The fourth scenario, if confirmed, could have important implications for the discussion about immunity against SARS-CoV-2. If some patients don’t develop antibodies, irrespective of factors such as age, sex or disease severity, are they nevertheless immune against reinfection, mediated e.g. by SARS-CoV-2 specific T-cells? Do the same effects apply to vaccination as well and if yes, how is the response to a possible vaccine best measured? These questions will have to be urgently answered before any sort of documentation of immunity against SARS-CoV-2 can be issued as has been suggested^20,21^.

In our cohort, neither the levels nor the general presence of anti-SARS-CoV-2 IgA and IgG against the S1-subunit of the spike protein determined via ELISA served as a predictor of disease severity as measured by the duration of hospitalization, treatment in the ICU or death, nor did we see an influence of gender or age on the antibody results. These findings confirm the results of previous studies^16,22^ but contradict the findings of others that tend to find higher levels of antibodies in men, older patients and more severely afflicted patients^17,23–27^. This discrepancy in the findings of different studies may be at least partially due to the different serological assays used, different makeups of the examined cohorts and different definitions of disease severity. In general, studies that examined neutralizing antibody titers found a correlation with disease severity, age and/or gender^22,24,26^, whereas only some studies using ELISA did^17,24,25,27^. Interestingly, the study by Tang et al. found a correlation between clinical severity and neutralizing antibody titers, but not for titers of antibodies detected via ELISA^22^, while the study by Klein et al. found this correlation for both neutralizing antibodies and ELISA antibodies^24^. In synopsis, while neutralizing antibodies clearly appear to be associated with disease severity, age and sex, this relationship is less clear for antibodies detected via ELISA. Of note, depending on the ELISA used, some studies found good correlations between neutralizing antibodies and antibodies detected via ELISA^28–30^, while others found that antibodies detected via ELISA reflected neutralizing activity only poorly^22,24^. Part of the explanation for this phenomenon is the fact that the titers of neutralizing antibodies have been found to decrease more sharply than antibody levels detected via ELISA^24,28^. Depending on the point in time since onset of symptoms the correlation between neutralization assay and ELISA may therefore be expected to vary with a better correlation at the peak of the antibody response. This is mirrored by the finding that commercial ELISAs (especially the one used by us) are particularly useful to detect individuals with high titers of neutralizing antibodies^29,30^. It is tempting to speculate that we didn’t find a correlation between antibody levels and clinical characteristics because the proportion of patients who had reached the peak level of their antibody response was low in our cohort. Inversely, this would mean that the peak titer of at least some commercial ELISAs might still be useful as a marker of disease severity and outcome, which is supported by the findings of Gudbjartsson et al.^17^, but more data is needed to confirm this hypothesis.

Finally we found higher age to be associated with a worse outcome, as measured by the significantly higher mean age of deceased patients as well as the case fatality rate per life decade. This result also supports earlier findings^31^ and stresses the important message that patients above the age of 60 should be considered to be at a higher risk of a complicated course of disease.

Our study has several limitations: Serum has been collected from each patient at a single point in time only, in many cases at a time that might have been too early for the detection of antibodies. In some of these individuals, a seroconversion might have been observed if the samples had been collected at a later stage or if follow-up examinations had taken place. We tried to minimize the effect of this potential bias by analyzing in a second step only those samples that were collected at least ten day after onset of symptoms (which is the amount of time for which the test properties are defined by the manufacturer). It is notable that we didn’t find any relevant differences between this subgroup and the whole cohort. Also, all samples were collected from hospitalized patients, potentially over representing more severe cases. However, the case-fatality rate of our cohort is 11% and therefore comparable to that of all of Spain (9.4%; CFR as of April 11th)^32^, suggesting that the severity of cases in our cohort are at least representative for the Spanish population from which they hail. Another limitation is that we tested for antibodies using only one target antigen as well as assays from only one manufacturer. It is possible that the patients for whom no anti-SARS-CoV-2 antibodies could be detected using our methods developed antibodies against other target antigens. It is noteworthy, however, that studies examining the antibody responses against different target antigens generally found a good correlation between them (as mentioned above)^17,18^. Furthermore, we did not confirm the results of our (commercial) ELISA with a neutralization assay. The relationship between antibody levels detected via ELISA and neutralization assays is complex and has been discussed at length above. In short, while it is likely that a portion of the antibodies we detected are not neutralizing, results of the ELISA we used have been shown to correlate well with the titers of neutralizing antibodies especially at high levels^24,29,30^, making it likely that a considerable share of the antibodies we detected were in fact neutralizing. Nevertheless, the results have to be interpreted with due caution.

Also, the PCR-confirmation of SARS-CoV-2 was performed with different assay. This was due to shortages of reagents for the SARS-CoV-2-rt-PCR that many laboratories experienced in the initial phase of the pandemic, forcing laboratories to diversify their assays.

In conclusion, our findings show that both the general detection of antibodies against SARS-CoV-2 and their levels depend largely on the time between onset of symptoms and sample collection and that neither the general presence of antibodies nor their levels can be used as a (prognostic) marker of disease severity. Due to the delay in the reliable detection of antibodies of two to three weeks since onset of symptoms, commercially available serologic assays have little to no value in suspected acute cases of COVID-19. Furthermore, our data appears to confirm that some patients don’t develop antibodies against SARS-CoV-2 within the usual time frame as well as it shows that those patients differ neither in demographic composition nor in disease severity from their anti-SARS-CoV-2 positive counterparts.

Questions still remain about possibly seronegative COVID-19 patients: what factors lead to a COVID-19 patient not developing antibodies (at least against the S1 subunit of the Spike-protein)? Also, our study did not examine how long detectable levels of antibodies persist in patients. Both of these questions are of importance for considerations of how and when herd-immunity against SARS-CoV-2 can be achieved and will have to be addressed in future studies.

## References

[1] Zhou P (2020). A pneumonia outbreak associated with a new coronavirus of probable bat origin. Nature.

[2] Chen N (2020). Epidemiological and clinical characteristics of 99 cases of 2019 novel coronavirus pneumonia in Wuhan, China: a descriptive study. Lancet Lond. Engl..

[3] Huang C (2020). Clinical features of patients infected with 2019 novel coronavirus in Wuhan, China. Lancet Lond. Engl..

[4] Wang D (2020). Clinical characteristics of 138 hospitalized patients with 2019 novel coronavirus-infected pneumonia in Wuhan, China. JAMA.

[5] Bajema KL (2020). Persons evaluated for 2019 novel coronavirus—United States, January 2020. MMWR Morb. Mortal. Wkly. Rep..

[6] Guan W-J (2020). Clinical characteristics of coronavirus disease 2019 in China. N. Engl. J. Med..

[7] Rajgor DD, Lee MH, Archuleta S, Bagdasarian N, Quek SC (2020). The many estimates of the COVID-19 case fatality rate. Lancet Infect. Dis..

[8] Corman VM (2020). Detection of 2019 novel coronavirus (2019-nCoV) by real-time RT-PCR. Euro Surveill. Bull..

[9] Solbach W (2020). Antibody profiling of COVID-19 patients in an urban low-incidence region in Northern Germany. medRxiv.

[10] Arvin AM (2020). A perspective on potential antibody-dependent enhancement of SARS-CoV-2. Nature.

[11] Jiang S, Hillyer C, Du L (2020). Neutralizing Antibodies against SARS-CoV-2 and other human coronaviruses. Trends Immunol..

[12] Zost SJ (2020). Potently neutralizing and protective human antibodies against SARS-CoV-2. Nature.

[13] Tang MS (2020). Association between SARS-CoV-2 neutralizing antibodies and commercial serological assays. bioRxiv.

[14] R Core Team (2020). R: A Language and Environment for Statistical Computing.

[15] Deeks JJ (2020). Antibody tests for identification of current and past infection with SARS-CoV-2. Cochrane Database Syst. Rev..

[16] Long Q-X (2020). Antibody responses to SARS-CoV-2 in patients with COVID-19. Nat. Med..

[17] Gudbjartsson DF (2020). Humoral immune response to SARS-CoV-2 in Iceland. N. Engl. J. Med..

[18] Isho B (2020). Persistence of serum and saliva antibody responses to SARS-CoV-2 spike antigens in COVID-19 patients. Sci. Immunol..

[19] Cervia C (2020). Systemic and mucosal antibody secretion specific to SARS-CoV-2 during mild versus severe COVID-19. bioRxiv.

[20] Phelan AL (2020). COVID-19 immunity passports and vaccination certificates: scientific, equitable, and legal challenges. Lancet Lond. Engl..

[21] Kofler N, Baylis FT (2020). reasons why immunity passports are a bad idea. Nature.

[22] Tang MS (2020). Association between SARS-CoV-2 neutralizing antibodies and commercial serological assays. Clin. Chem..

[23] Madariaga MLL (2020). Clinical predictors of donor antibody titer and correlation with recipient antibody response in a COVID-19 convalescent plasma clinical trial. J. Intern. Med..

[24] Klein S (2020). Sex, age, and hospitalization drive antibody responses in a COVID-19 convalescent plasma donor population. MedRxiv Prepr. Serv. Health Sci..

[25] Zhao J (2020). Antibody responses to SARS-CoV-2 in patients of novel coronavirus disease 2019. Clin. Infect. Dis..

[26] Seow J (2020). Longitudinal evaluation and decline of antibody responses in SARS-CoV-2 infection. MedRxiv.

[27] Marklund E (2020). Serum-IgG responses to SARS-CoV-2 after mild and severe COVID-19 infection and analysis of IgG non-responders. PLoS ONE.

[28] Beaudoin-Bussières G (2020). Decline of humoral responses against SARS-CoV-2 spike in convalescent individuals. mBio.

[29] Patel E (2020). Comparative performance of five commercially available serologic assays to detect antibodies to SARS-CoV-2 and identify individuals with high neutralizing titers. MedRxiv Prepr. Serv. Health Sci..

[30] Wajnberg A (2020). Robust neutralizing antibodies to SARS-CoV-2 infection persist for months. Science.

[31] Verity R (2020). Estimates of the severity of coronavirus disease 2019: A model-based analysis. Lancet Infect. Dis..

[32] Roser, M., Ritchie, H., Ortiz-Ospina, E. & Hasell, J. Coronavirus Pandemic (COVID-19). *Our World Data* (2020).

